# Supplementary material for: Enteral Activation of WR-2721 Mediates Radioprotection and Improved Survival from Lethal Fractionated Radiation
Source: Sci Rep. 2019 Feb 13;9:1949. doi: 10.1038/s41598-018-37147-9 (PMC6374382; doi:10.1038/s41598-018-37147-9)
Supplement: Supplementary file 1 — Figure S1 [file 41598_2018_37147_MOESM1_ESM.pdf]

Enteral Activation of WR-2721 Mediates Radioprotection and Improved Survival from Lethal Fractionated Radiation

Jessica M. Molkentine, Tara N. Fujimoto, Thomas D. Horvath, Aaron J. Grossberg, Carolina J. Garcia Garcia, Amit Deorukhkar, Marimar de la Cruz Bonilla, Daniel Lin, Errol L.G. Samuel, Wai Kin Chan, Philip L. Lorenzi, Helen Piwnica-Worms, Robert Dantzer, James M. Tour, Kathryn A. Mason, Cullen M. Taniguchi

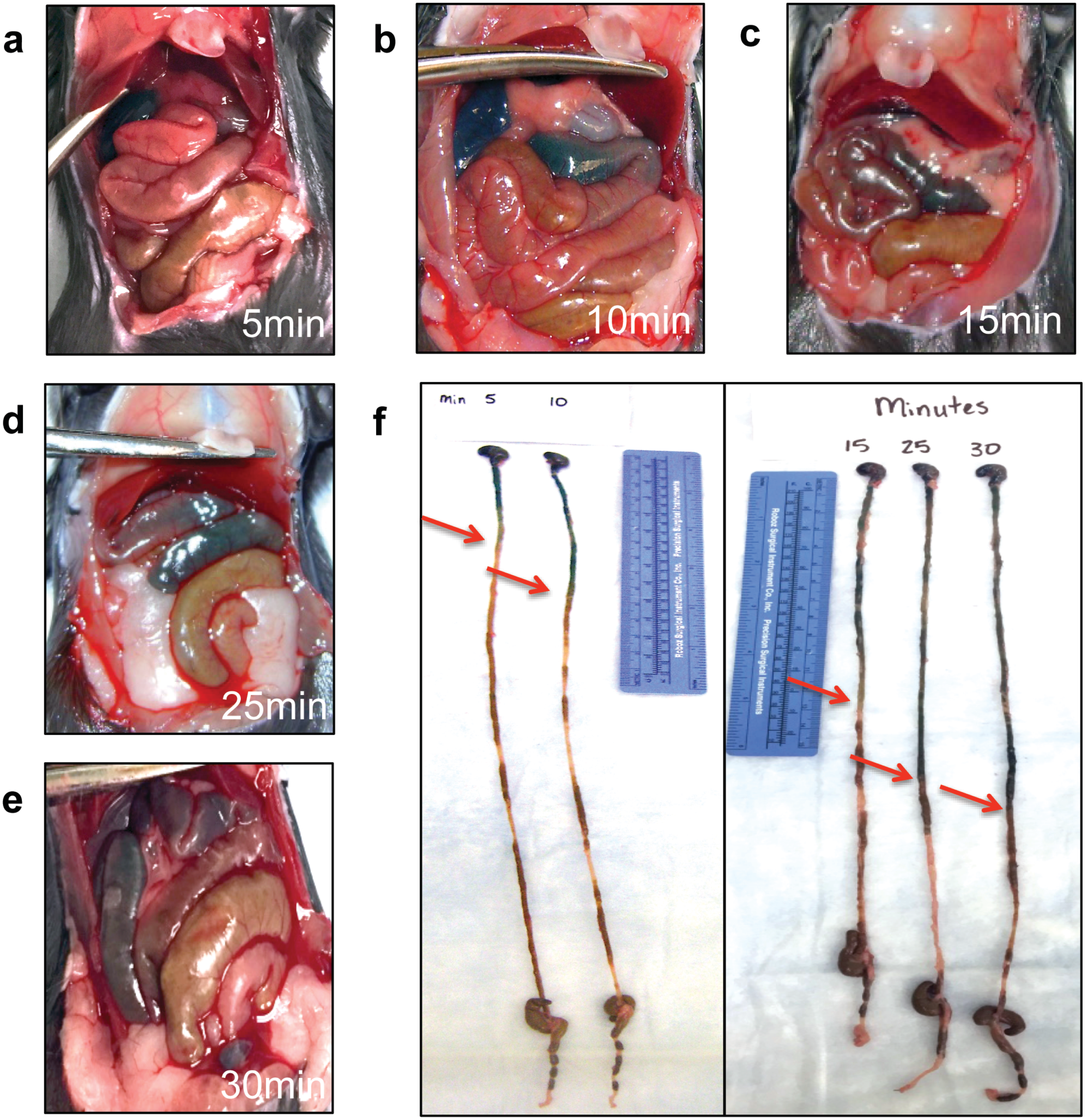

Figure S1. GI transit time following oral administration of methylene blue (a-e) Progress of methylene blue dye within the GI tract in situ following oral gavage at 5 min (a), 10 min (b), 15 min (c), 25 min (d) and 30 min (e) time points. **(f)** Comparison of resected GI tract from stomach to colon from the same mice shown in (a-e). Red arrows indicate the dye front.
